# A novel initial wiring technique for chronic total occlusion of the superficial femoral artery using the structural features of a polymer jacket guidewire

**DOI:** 10.1186/s42155-022-00313-2

**Published:** 2022-07-21

**Authors:** Eiji Karashima, Yoshimitsu Soga, Takeshi Arima, Hirotaka Noda, Shioto Yasuda, Takeo Kaneko

**Affiliations:** 1 Department of Cardiology, Shimonoseki City Hospital, 1-13-1 Kouyou-chou, Shimonoseki, Yamaguchi 750-8520 Japan; 2 Department of Cardiology, Kokura Memorial Hospital, Kitakyushu, Japan

**Keywords:** Chronic total occlusion, Endovascular therapy, Femoropopliteal artery disease, Peripheral arterial disease

## Abstract

**Background:**

To evaluate the efficacy of the GLadIus MG drilLINg technique (GLIMGLIN), a novel initial wiring technique using the Gladius MG™ structural features, for crossing the superficial femoral artery (SFA) with chronic total occlusion (CTO).

**Methods:**

This retrospective, single-center study enrolled 27 symptomatic patients (mean age 77.4 ± 8.5 years; 20 men) with de novo SFA CTO (mean CTO length 16.1 ± 8.9 cm) who underwent GLIMGLIN as the initial wiring between January 2020 and December 2021. The success of GLIMGLIN was defined when the wire crossing was completed using a Gladius MG™ and a microcatheter without any additional devices and techniques.

**Results:**

The success rate of GLIMGLIN was 48.1%. Intravascular ultrasound findings showed complete true lumen passage in the GLIMGLIN success group. Compared to the failure group, the proximal (6.3 ± 0.8 vs. 5.5 ± 0.9 mm, *p* = 0.02) and distal (5.9 ± 0.5 vs. 5.4 ± 0.6 mm, *p* = 0.02) reference vessel diameters were significantly larger, and the rate of calcium angle > 180° was significantly lower (30.8 vs. 71.4%, *p* = 0.04) in the success group. No significant difference was shown in the CTO length between two groups. Total wiring time, total procedure time, and fluoroscopic time were significantly shorter in the success group.

**Conclusions:**

GLIMGLIN may enable operators to perform CTO wiring easily and efficiently in selected cases.

**Supplementary Information:**

The online version contains supplementary material available at 10.1186/s42155-022-00313-2.

## Background

Endovascular therapy (EVT) has evolved as a first-line treatment for superficial femoral artery (SFA) occlusive disease (Norgren et al. 2007; Aboyans et al. 2018). However, wire crossing in patients with chronic total occlusion (CTO) of the SFA can be challenging in case of long lesion length, calcification, difficulty in penetrating the proximal and distal caps, and inadvertent subintimal passage of crossing devices. Conventional wiring technique, with drilling and rotation of wires supported by microcatheters, is the initial and most frequently used technique for crossing the SFA CTO. However, it is often difficult to avoid unintentional subintimal passage during conventional wiring technique. Additionally, wire crossing with conventional wiring technique can fail in 50% of infrainguinal CTO (Banerjee et al. 2015). Although the success of CTO wiring with conventional wiring technique is usually based on technical training and personal experience of the operator, the development of new device scan helps avoiding a recanalization through the subintimal space. Therefore, we reported the GLadIus MG drilLINg technique (GLIMGLIN), a novel wiring technique using the Gladius MG™ structural feature, that could avoid wire advancement into the subintimal lumen.

**Fig. 1:**
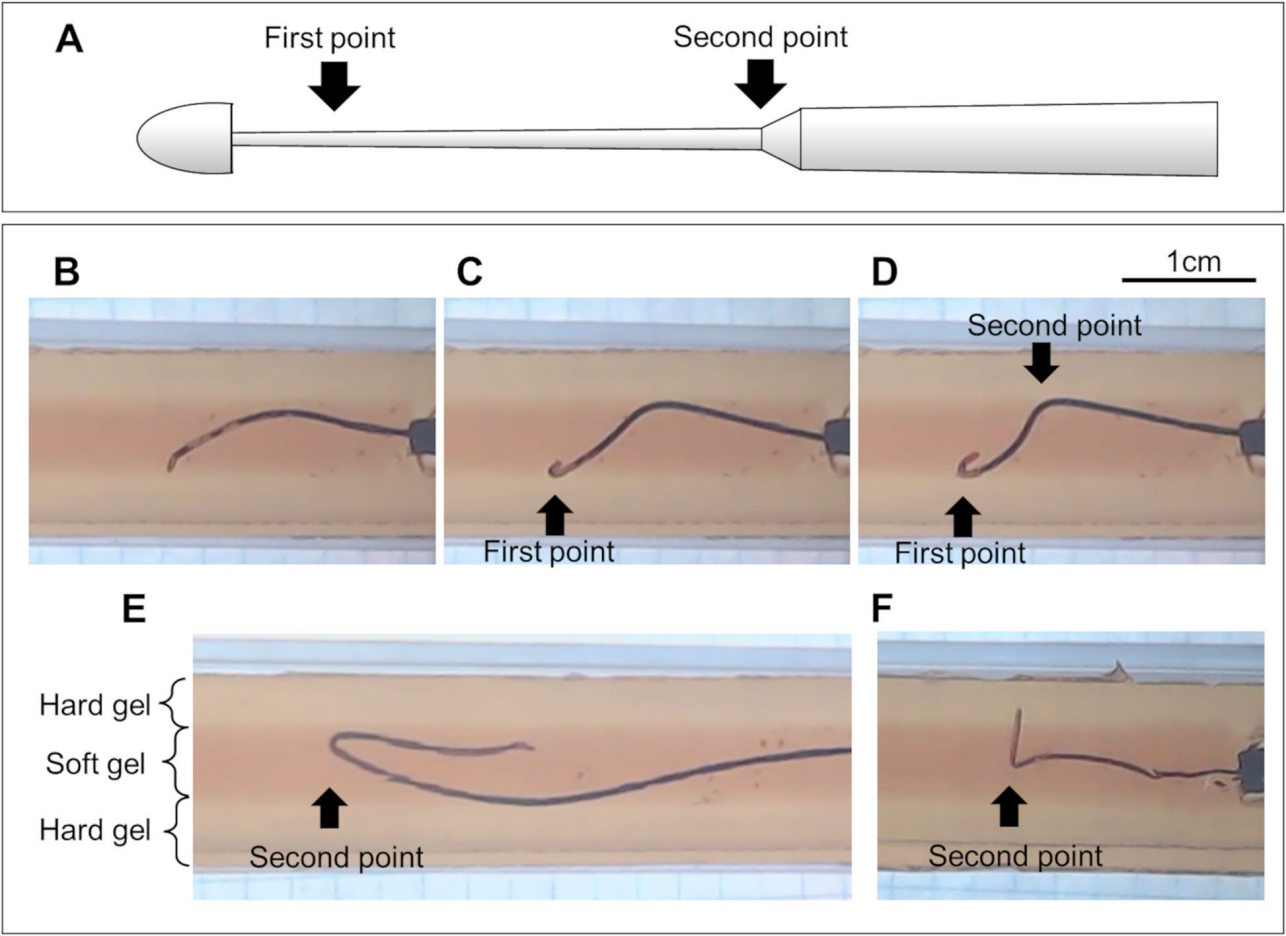
Scheme of the round core and pictures in the CTO model of Gladius MG™. **a** Because the tip of the round core is narrowing, the Gladius MG™ has two bending points. (B-E) A 0.018-inch Gladius MG™ was inserted into the soft gel of the CTO model. **b** The tip of the Gladius MG™ was attached to the hard gel. **c** The Gladius MG™ was pushed further after the tip was attached to the hard gel; the first point was bending. **d** The Gladius MG™ was pushed further after being bent the first point, the second point began to bend. **e** When the Gladius MG™ was pushed more after bending at the second point, the Gladius MG™ was advanced into the soft gel with a looped shape. The bending point of the looped shape Gladius MG™ was the second point. **f** When the tip and the first point of the 0.014-inch Gladius MG™ advanced into the hard gel, on pushing the wire further, the second point began to bend

Gladius MG™ (Asahi Intecc, Aichi, Japan) is a polymer jacket guidewire. The sizes of Gladius MG14 and 18 PVES were 0.014 and 0.018 inches, respectively. The tip load of Gladius MG™ is 3 g, the tip of the round core is narrowing, and the Gladius MG™ has two bending points (Fig. 1a). When the first point is bending, the wire is considered to attach to the vessel wall or stiff lesion. When the wire is pushed further, additional bending begins at the second point because the shaft hardness of the Gladius MG™ suddenly increases at the second point.

GLIMGLIN is a novel wiring technique that uses the structural features of Gladius MG™. When the Gladius MG™ bent at the first or second bending point, the wire was pulled back to avoid wire advancement into the subintimal space. This study aimed to investigated the efficacy of GLIMGLIN as an initial wiring technique for SFA CTO. Additionally, to investigate the structural features of Gladius MG™, which may help understand how the wire advancement into the subintimal space during GLIMGLIN could be avoided, we performed the experimental study of the Gladius MG™ wiring in the CTO model.

## Methods

### Experimental study

A preliminary experiment of wiring using the Gladius MG™ was performed in a CTO model (Fig. 1). The CTO model consisted of layers of soft and hard gels. A soft gel to replicate a soft plaque was surrounded by a hard gel that replicates the vessel wall. A 0.018-inch Gladius MG™ (Gladius MG18 PVES) was inserted into the soft gel. After the wire tip touched the hard gel (Fig. 1b), the wire bent at the first point (Fig. 1c). As the wire was pushed further, the second point began to bend (Fig. 1d). Figure 1e showed the shape of a 0.018-inch Gladius MG™ during the narrowing-loop wire technique in the soft gel. The second point of the Gladius MG™ was bent during the narrowing-loop wire technique. Figure 1f showed the shape of a 0.014-inch Gladius MG™ when the tip and the first point advanced into the hard gel. As the wire was pushed further, the second point began to bend.

### Clinical study

**Fig. 2:**
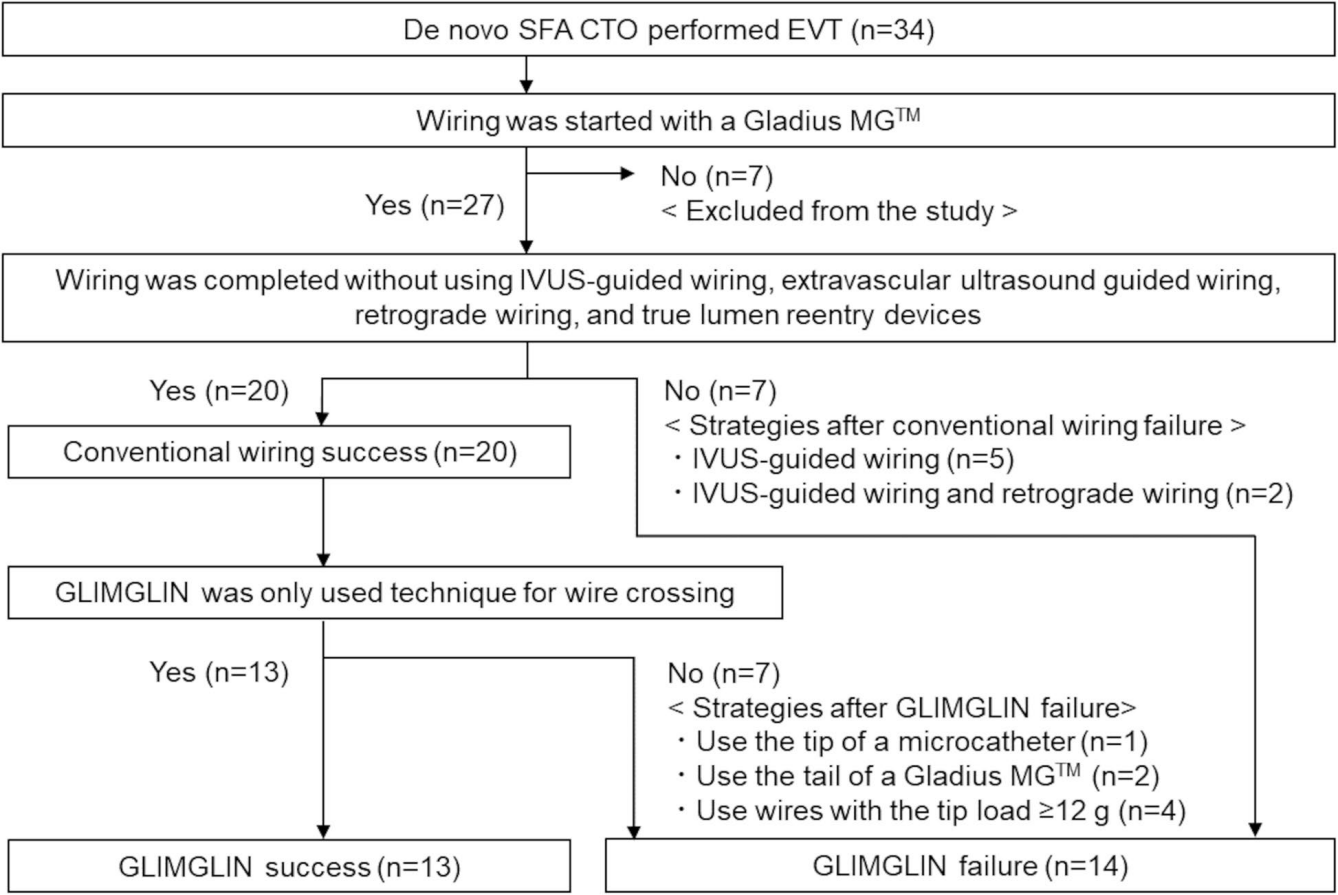
The study flowchart. During the study, the 34 symptomatic PAD patients with de novo SFA CTO performed EVT, and the 27 patients were included to the study. In those, conventional wiring and GLIMGLIN were succeeded in 20 and 13 patients, respectively

A retrospective single-center study was performed to analyze differences in patient, lesion, and procedural characteristics between the success and failure groups of GLIMGLIN in patients with SFA EVT conducted between January 2020 and December 2021. Patients with in-stent restenosis or acute limb ischemia were excluded from the study. Using GLIMGLIN as the initial wiring for SFA CTO was at the operator’s discretion. Figure 2 showed the study flowchart. When a Gladius MG™ was selected as the first wire of the CTO wiring, because GLIMGLIN was always performed in such cases, the patients were enrolled to the study. Conventional wiring success was defined when the CTO wiring was completed using wires and microcatheters without intravascular ultrasound (IVUS)-guided wiring, extravascular ultrasound guided wiring, retrograde wiring, and true lumen reentry devices. GLIMGLIN success was defined when the CTO wiring was completed using a Gladius MG™ and a microcatheter without any additional devices and techniques in cases of conventional wiring success. Therefore, GLIMGLIN was defined as one of the techniques of the conventional wiring.

Outcome measures included GLIMGLIN success, wire crossing success, procedure success, and major adverse events (MAEs) within 30 days. Procedure success was defined as successful revascularization of the SFA CTO with ≤ 30% angiographic residual diameter stenosis. MAEs included all-cause mortality, nonfatal myocardial infarction, ischemic stroke, and unplanned revascularization or amputation of the target limb. Wiring time was defined from the time when a wire reached to the proximal CTO to the time when the wire passage was confirmed by IVUS or contrast injection to the CTO distal segments from a microcatheter.

The study protocol was developed following the Declaration of Helsinki and approved by the hospital’s ethics committee. All patients provided written informed consent for EVT.

### Intervention protocols

**Fig. 3:**
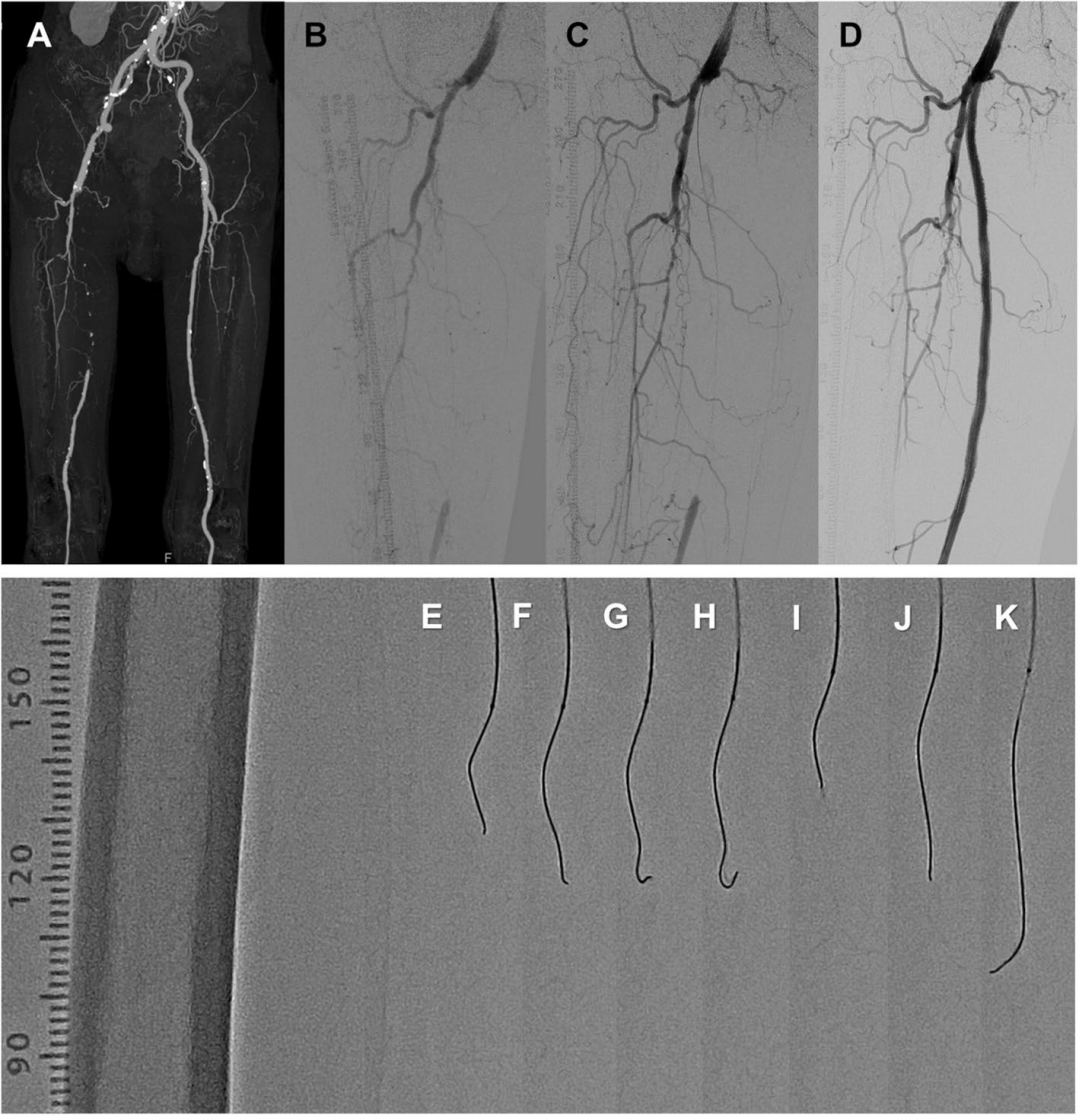
A representative case of GLIMGLIN. **a** A computed tomographic angiography revealed a right SFA CTO. Angiography of pre-EVT (**b**), after the wire crossing (**c**), and post EVT (**d**). **e-k** Shapes of a 0.018-inch Gladius MG™ during GLIMGLIN. When the first point was bending (**g, h**), the wire pulled back (**i**). The wire was rotated to select a different route for its advancement without bending at the first and second points (**j, k**)

Three experienced physicians performed all procedures. After insertion of a 6-F sheath from the common femoral artery, unfractionated heparin (5000 units) was injected from the sheath, and 1000 units of heparin were routinely added on an hourly basis. The wiring with GLIMGLIN was performed with a Gladius MG™ supported by a microcatheter. A representative case of successful wiring using GLIMGLIN was shown in Fig. 3. The shapes of the 0.018-inch Gladius MG™ (Gladius MG18 PVES) during GLIMGLIN were shown in Fig. 3e to 3k. When the first point was bending (Fig. 3g and h), the wire was pulled back (Fig. 3i). The wire was rotated to select a different route in which the wire could advance without bending at the first and second point (Fig. 3j and 3k). In this case, the CTO length was 21 cm, and the total wiring time was 4 min. During this study, GLIMGLIN was continued when the Gladius MG™ could advance through the lesion. However, when the Gladius MG™ could not advance at a point of the lesion for more than 3 min, the GLIMGLIN was abandoned. When the GLIMGLIN was abandoned, the wire was changed, or different techniques were used to cross the CTO lesion. Therefore, these cases were categorized as the failure group. After wire crossing of the CTO lesion, an IVUS catheter was employed to investigate the wire route, proximal and distal external elastic membranes (EEM), and maximum calcium angle of the CTO lesion.

### Statistical analysis

Continuous data were presented as means ± standard deviations, and categorical data were presented as numbers (percentages). As appropriate, continuous variables between the groups were compared using an unpaired t-test or Mann–Whitney U test. Categorical data were compared using the Chi-squared test. Statistical analyses were performed using SPSS version 27 (IBM Corporation, Armonk, NY, USA). Statistical significance was set at *p* < 0.05.

## Results

### Results of the experimental study

In the CTO model, the first and second points of a 0.018-inch Gladius MG™ were approximately 2 mm and 12 mm from the tip, respectively (Fig. 1d and e). The length from the tip to the second point of a 0.018-inch Gladius MG™ was longer than that of a 0.014-inch Gladius MG™ (Fig. 1e and f). After the wire tip touched the hard gel, the wire bent at the first point (Fig. 1c) or advanced into the hard gel (Fig. 1f). As the wire was pushed further, the second point began to bend (Fig. 1d and f).

### Results of the clinical study

During the study, EVT was performed in 273 symptomatic patients with peripheral arterial disease (PAD) in our hospital. As shown in Fig. 2, de novo SFA CTO was detected in 34 patients, and EVT was performed. Of these, wiring was started with a Gladius MG™ in 27 patients (mean age: 77.4 ± 8.5 years; 20 male). Because a Gladius MG™ was not used as the first wire of CTO wiring, 7 patients were excluded. In the 27 participants, the conventional wiring success was shown in 20 patients (74.1%). After the conventional wiring failure, 7 patients were categorized into the GLIMGLIN failure group, the IVUS-guided wiring was always performed. In these cases, retrograde wiring was also performed in 2 patients. For patients with conventional wiring success, GLIMGLIN was successful in 13 patients (success group, mean age: 74.5 ± 7.6 years; 11 male). Therefore, even when conventional wiring was successful, GLIMGLIN failed in 7 patients. In these cases, the lesions, which could not be crossed by a Gladius MG™, were crossed with the tip of a microcatheter (*n* = 1), tail of a Gladius MG™ (n = 2), and the wires with the tip load ≥ 12 g (*n* = 4), respectively. Because 14 patients were categorized as the GLIMGLIN failure group (mean age: 80.0 ± 8.6 years; 9 male), the success rate of GLIMGLIN was 48.1%. The GLIMGLIN success and failure groups patient, lesion, and procedural characteristics were summarized in Tables 1 and 3, respectively. The patient characteristics showed no significant difference between two groups, except that more patients in the failure group had dyslipidemia.

**Table 1:** Patient characteristics

	All (*n* = 27)	GLIMGLIN		*P*
		Success (*n* = 13)	Failure (*n* = 14)	
Age, years	77.4 ± 8.5	74.5 ± 7.6	80.0 ± 8.6	0.14
Male, n (%)	20 (74.1)	11 (84.6)	9 (64.3)	0.24
Body mass index, kg/m^2^	21.9 ± 3.7	21.5 ± 4.0	22.3 ± 3.4	0.42
Hypertension, n (%)	26 (96.3)	12 (92.3)	14 (100)	0.30
Dyslipidemia, n (%)	15 (55.6)	4 (30.8)	11 (78.6)	0.01*
Diabetes, n (%)	13 (48.1)	6 (46.2)	7 (50.0)	0.85
Smoking, n (%)	25 (92.6)	12 (92.3)	13 (92.9)	0.96
Coronary artery disease, n (%)	8 (29.6)	4 (30.8)	5 (35.7)	0.79
Cerebrovascular disease, n (%)	7 (25.9)	2 (15.4)	5 (35.7)	0.24
Atrial fibrillation, n (%)	7 (25.9)	3 (23.1)	4 (28.6)	0.75
Heart failure, n (%)	8 (29.6)	4 (30.8)	4 (28.6)	0.90
eGFR < 60, n (%)	18 (66.7)	8 (61.5)	10 (71.4)	0.59
Haemodialysis, n (%)	3 (11.1)	1 (7.7)	2 (14.3)	0.59
Rutherford classification				
2,3	13 (48.1)	8 (61.5)	6 (42.9)	0.34
4	7 (25.9)	1 (7.7)	5 (35.7)	0.09
5,6	7 (25.9)	4 (30.8)	3 (21.4)	0.59

^*^P < 0.05, Values are expressed as mean ± standard deviation or number (percentage)

The lesion characteristics were shown in Table 2. For angiography, the proximal (6.3 ± 0.8 vs. 5.5 ± 0.9 mm, *p* = 0.02) and distal (5.9 ± 0.5 vs. 5.4 ± 0.6 mm, *p* = 0.02) reference vessel diameters (RVD) were significantly larger in the success group than in the failure group. In contrast, the lesion length, CTO length, peripheral arterial calcium scoring system (PACSS) grade (Rocha-Singh et al. 2014), and the number of the below-the-knee run-offs showed no significant difference between two groups. IVUS findings showed complete, true lumen passage in the success group. Additionally, the rate of calcium angle > 180° detected by IVUS was significantly fewer (30.8 vs. 71.4%, p = 0.04) in the success group than in the failure group. Even if the proximal and distal RVDs were significantly larger in the success group than in the failure group, no significant difference was observed in the proximal and distal EEM between two groups.

**Table 2:** Lesion characteristics

	All (*n* = 27)	GLIMGLIN		*P*
		Success (*n* = 13)	Failure (*n* = 14)	
Angiography findings				
Lesion length (cm)	23.6 ± 8.0	23.3 ± 7.5	23.9 ± 8.8	0.73
CTO length (cm)	16.1 ± 8.9	15.6 ± 8.4	16.6 ± 9.7	0.80
CTO < 5 cm	4 (14.8)	2 (15.4)	2 (14.3)	0.98
CTO 5 ~ 15 cm	9 (33.3)	5 (38.5)	4 (28.6)	0.06
CTO > 15 cm	14 (51.9)	6 (46.2)	8 (57.1)	0.58
Proximal RVD	5.9 ± 0.9	6.3 ± 0.8	5.5 ± 0.9	0.02*
Distal RVD	5.6 ± 0.6	5.9 ± 0.5	5.4 ± 0.6	0.02*
PACSS grade				
0	12 (44.4)	8 (61.5)	4 (28.6)	0.09
1	4 (14.8)	1 (7.7)	3 (21.4)	0.33
2	5 (18.5)	1 (7.7)	4 (28.6)	0.17
3	2 (7.4)	1 (7.7)	1 (7.1)	0.96
4	4 (14.8)	2 (15.4)	2 (14.3)	0.94
Below-the-knee run-off				
0	4 (14.8)	2 (15.4)	2 (14.3)	0.94
1	5 (18.5)	4 (30.8)	1 (7.1)	0.12
2	11 (40.7)	4 (30.8)	7 (50.0)	0.32
3	7 (25.9)	3 (23.1)	4 (28.6)	0.75
IVUS findings				
Complete true lumen passage	22 (81.5)	13 (100)	9 (64.3)	0.02*
Proximal EEM	39.1 ± 9.6	39.1 ± 11.1	39.0 ± 8.4	0.88
Distal EEM	29.6 ± 9.7	29.3 ± 9.0	29.8 ± 10.6	0.92
Calcium angle				
0 ~ 90°	8 (29.6)	5 (38.5)	3 (21.4)	0.34
91 ~ 180°	5 (18.5)	4 (30.8)	1 (7.1)	0.12
181 ~ 270°	8 (29.6)	2 (15.4)	6 (42.9)	0.13
271 ~ 360°	6 (22.2)	2 (15.4)	4 (28.6)	0.42
Calcium angle > 180°	14 (51.9)	4 (30.8)	10 (71.4)	0.04*

^*^P < 0.05, Values are expressed as mean ± standard deviation or number (percentage)

The procedural characteristics and short-term outcomes within 30 days were presented in Table 3. The number of wires for crossing were significantly smaller in the success group. Additionally, for the wire crossing, distal puncture and IVUS-guided wiring were not performed in the success group. The total wiring time, total procedure time, and fluoroscopic time were significantly longer in the failure group than in the success group. Even though the Rutherford category distribution at 30 days showed no significant difference between two groups, the post-ankle brachial index was significantly lower in the failure group than in the success group.

**Table 3:** Procedural characteristics

	All (*n* = 27)	GLIMGLIN		*P*
		Success (*n* = 13)	Failure (*n* = 14)	
Access				
Ipsilateral	23 (85.2)	13 (100)	10 (71.4)	0.04*
Contralateral	4 (14.8)	0 (0.0)	4 (28.6)	0.04*
Size of Gladius MG™				
0.014 inch	12 (44.4)	5 (38.5)	7 (50.0)	0.55
0.018 inch	15 (55.6)	8 (61.5)	7 (50.0)	0.55
Wiring time				
GLIMGLIN (min)	7.1 ± 6.4	6.2 ± 5.6	7.9 ± 7.2	0.83
Total wiring time (min)	30.5 ± 35.3	6.2 ± 5.6	53.0 ± 36.5	< 0.001*
Wire crossing				
Wire crossing success (%)	27 (100)	13 (100)	14 (100)	> 0.99
Conventional wiring success (%)	20 (74.1)	13 (100)	7 (50.0)	0.03*
Number of wires for crossing	1.9 ± 1.1	1.0 ± 0.0	2.7 ± 1.0	< 0.001*
IVUS-guided wiring (%)	7 (25.9)	0 (0.0)	7 (50.0)	0.03*
Distal puncture (%)	2 (7.4)	0 (0.0)	2 (14.3)	0.55
Total procedure time (min)	103.4 ± 35.4	80.2 ± 21.7	125.0 ± 32.1	< 0.001*
Total contrast volume (ml)	51.1 ± 16.7	45.8 ± 13.8	56.0 ± 18.1	0.15
Fluoroscopic time (min)	36.9 ± 21.1	23.6 ± 6.9	49.2 ± 22.4	< 0.001*
Procedural success (%)	27 (100)	13 (100)	14 (100)	> 0.99
Final device				
Drug coated balloon	14 (51.9)	5 (38.5)	9 (64.3)	0.19
Bare nitinol stent	3 (11.1)	2 (15.4)	1 (7.1)	0.50
Drug eluting stent	9 (33.3)	6 (46.2)	3 (21.4)	0.18
Interwoven stent	1 (3.7)	0 (0.0)	1 (7.1)	0.34
Pre ABI	0.51 ± 0.14	0.50 ± 0.18	0.53 ± 0.11	0.80
Post ABI	0.82 ± 0.23	0.93 ± 0.20	0.73 ± 0.22	0.02*
Short term outcomes within 30 days				
All cause death	0 (0.0)	0 (0.0)	0 (0.0)	> 0.99
Major adverse events	1 (3.7)	0 (0.0)	1 (7.1)	0.36
Major amputation	1 (3.7)	0 (0.0)	1 (7.1)	0.36
Minor amputation	1 (3.7)	1 (7.7)	0 (0.0)	0.28
TLR within 30 days	0 (0.0)	0 (0.0)	0 (0.0)	> 0.99
Rutherford classification at 30 days				
0,1	20 (74.1)	9 (69.2)	11 (78.6)	0.59
2,3	1 (3.7)	0 (0.0)	1 (7.1)	0.34
4	0 (0.0)	0 (0.0)	0 (0.0)	> 0.99
5,6	6 (22.2)	4 (30.8)	2 (14.3)	0.31

^*^P < 0.05, Values are expressed as mean ± standard deviation or number (percentage)

## Discussion

GLIMGLIN, a novel initial wiring technique using the Gladius MG™ structural features, was useful to avoid the wire advancement into the subintimal space during SFA CTO wiring. Bending at the first point was considered to represent the attachment of the tip to the hard plaque or vessel wall. Additionally, bending at the second point was considered to represent the attachment of the tip to or its advancement into the hard plaque or vessel wall. Additional wiring with different wires was started after the failure of GLIMGLIN, it was acceptable that the total wiring time, total procedure time, and fluoroscopic time were significantly longer in the failure group than in the success group.

The narrowing-loop wire technique also use the structural features of Gladius MG™ and was reported as a useful wiring technique (Nakama et al. 2020). However, since the wire can advance into the subintimal space, the narrowing-loop wire technique was not used in this study.

Even if the conventional wiring is the most frequently used wiring technique, avoiding the wire advancement into the subintimal space is difficult. To overcome the difficulty of recanalization through the subintimal space, retrograde wiring, trans-collateral wiring, IVUS-guided wiring, and the use of a true lumen reentry device have been reported (Schmidt et al. 2012; Urasawa et al., 2014; Kitrou et al. 2015; Tan et al. 2017; Hayakawa et al. 2021; Tan et al. 2022). Although exceptional skills and additional devices are needed, these techniques are quite effective in crossing the wire into CTO lesions. However, avoiding wire advancement into the subintimal space is not the purpose of these techniques.

Compared to 0.014- or 0.018-inch wires, the 0.035-inch wires generally have greater rail support and can be used to improve torque, whereas its flexibility and trackability through tortuous segment are smaller. In this study, we could not compare the efficacy of 0.035-inch wires because the size of Gladius MG™ were limited to 0.014 and 0.018 inches.

The “leave nothing behind” strategy, an aggressive lesion preparation followed by drug coated balloons without bailout stenting requirement, considered one of the ideal procedures. This approach was attempted for most SFA lesions in our hospital during this study. An intraluminal recanalization can improve the chance to treat the SFA CTO with the "leave nothing behind" strategy because the intraluminal recanalization improved the clinical outcomes of EVT for SFA CTO (Tsubakimoto et al. 2021). During this study, even when the intraluminal wiring was confirmed, the use of drug coated balloons as the final device of EVT did not increase in the GLIMGLIN success group. Our results suggested that only the intraluminal wiring was insufficient to treat the SFA CTO with the "leave nothing behind" strategy.

Several studies reported that the success rates of wire crossing with the conventional wiring technique for CTO with a mean length ≥ 10 cm were 33.3% to 65.8% (Charalambous et al. 2010; Shetty et al. 2013; Banerjee et al. 2015). However, in the success group of conventional wiring, the number of wires was not described, and the rate of true lumen passage was not completely confirmed by IVUS in those studies. In contrast, only one wire of the Gladius MG™ was required during GLIMGLIN. Moreover, as shown in Fig. 2 and Table 3, GLIMGLIN was performed and the wire crossing was succeeded in all of the 27 participants, conventional wiring success was shown in 20 patients (74.1%) even when the CTO length was 16.1 ± 8.9 cm. Therefore, GLIMGLIN might be an easier method for the initial SFA CTO wiring.

In this study, we investigated the lesion and procedural characteristics in the success group. Of the lesion characteristics, the CTO length was not associated with the success rate of GLIMGLIN. However, vessel size and lesion calcification were associated with the success rate of GLIMGLIN. Indeed, for the vessel size investigation, the proximal and distal RVDs were significantly larger in the success group than in the failure group. For the investigation of lesion calcification, a calcium angle > 180° was significantly lower in the success group than in the failure group. Additionally, although no significant difference was observed in the PACSS grade between the groups, most of the success cases (n = 8, 61.5%) had PACSS grade 0. Regarding the procedure characteristics, GLIMGLIN failed in cases where the contralateral approach was performed. Therefore, it was considered difficult to use the structural features of Gladius MG™ in the contralateral approach. These findings suggested that larger vessels and less calcified lesions with the ipsilateral approach were more suitable procedures for GLIMGLIN.

### Limitations

This study had several limitations. First, during GLIMGLIN, the wire was limited to Gladius MG™. Second, during the study, GLIMGLIN was limited to the initial wiring of the SFA CTO. Therefore, we could not compare the success rates of conventional wiring with different wires and GLIMGLIN. Third, while the complete true lumen passage was shown in all cases with successful GLIMGLIN in this study, conclusive evidence of the prevention of wire advancement into the subintimal space by GLIMGLIN was not provided. Fourth, even though the tip load of the Gladius MG™ was 3 g, we gave up the wiring with GLIMGLIN when the Gladius MG™ could not advance at all in a point of the lesion. Finally, the number of patients in this study was small. Further accumulation of experience and investigation are needed to confirm the preliminary results of our study.

## Conclusions

We presented the efficacy of GLIMGLIN, one of the conventional wiring techniques, as an initial wiring technique for SFA CTO. The success rate of GLIMGLIN for the mean CTO length of 16.1 ± 8.9 cm was 48.1%, and the IVUS findings showed complete true lumen passage in the success group. Large vessels, less calcified lesions, and the ipsilateral approach were considered suitable for GLIMGLIN. GLIMGLIN may enable operators to perform CTO wiring easily and efficiently in selected cases.

## Supplementary Information

**Additional file 1: Movie 1.** Movieof wiring experiments of Gladius MG^TM^ in the CTO model.

**Additional file 2: Movie 2.** Angiographyof the representative case of successful SFA CTO wiring using GLIMGLIN.

## Data Availability

The datasets used and/or analyzed during this study are available from the corresponding author on reasonable request.

## Abbreviations

ABI: Ankle brachial index
CTO: Chronic total occlusion
EEM: External elastic membrane
eGFR: Estimated glomerular filtration rate
EVT: Endovascular therapy
GLIMGLIN: GLadIus MG drilLINg technique
IVUS: Intravascular ultrasound
MAE: Major adverse event
PACSS: Peripheral arterial calcium scoring system
PAD: Peripheral arterial disease
RVD: Reference vessel diameter
SFA: Superficial femoral artery
TLR: Target lesion revascularization
